# Performance of location-scale models in meta-analysis: A simulation study

**DOI:** 10.3758/s13428-025-02622-5

**Published:** 2025-03-17

**Authors:** Desirée Blázquez-Rincón, José Antonio López-López, Wolfgang Viechtbauer

**Affiliations:** 1 Department of Psychology, Universidad a Distancia de Madrid, Madrid, Spain; 2 Department of Basic Psychology and Methodology, University of Murcia, Murcia, Spain; 3 Department of Psychiatry and Neuropsychology, Maastricht University, Maastricht, The Netherlands

**Keywords:** Location-scale models, Meta-analysis, Heterogeneity, Meta-regression, Moderator analysis

## Abstract

Location-scale models in the field of meta-analysis allow researchers to simultaneously study the influence of moderator variables on the mean (location) and variance (scale) of the distribution of true effects. However, the increased complexity of such models can make model fitting challenging. Moreover, the statistical properties of the estimation and inference methods for such models have not been systematically examined in the meta-analytic context. We therefore conducted a Monte Carlo simulation study to compare different estimation methods (maximum or restricted maximum likelihood estimation), significance tests (Wald-type, permutation, and likelihood-ratio tests), and methods for constructing confidence intervals (Wald-type and profile-likelihood intervals) for the scale coefficients of such models. When restricted maximum likelihood estimation was used, slightly closer to nominal rejection rates and narrower confidence intervals were obtained. The permutation test yielded type I error rates closest to the nominal level, whereas the likelihood-ratio test obtained the highest statistical power. In most scenarios, profile-likelihood intervals showed lower coverage probabilities than the Wald-type method but closer to the nominal 95% level. Finally, slightly higher rejection rates and coverage probabilities were obtained when a dichotomous moderator was examined rather than a continuous one. Despite the need to use some constraints on the parameter space for the scale coefficients and the possibility of non-convergence of some procedures that may affect the fitting of the specified models, location-scale models proved to be a valid and useful tool for modeling the heterogeneity parameter in meta-analysis.

**Supplementary information:**

The online version contains supplementary material available at 10.3758/s13428-025-02622-5.

## Introduction

Meta-analysis is a research tool that allows for the quantitative integration of the empirical evidence collected from several studies aimed at examining the same phenomenon (Cooper et al., 2019). Regardless of the nature of the phenomenon of interest, a primary goal in a meta-analysis is to examine the average effect or outcome. To do so, commensurable evidence is obtained from the studies in the form of an effect size and, following, a pooled effect is computed based on a particular statistical model. Two models are often used in practice, the fixed-effect and the random-effects model (Borenstein et al., 2010), the former being a special case of the latter. Next, we briefly introduce the basis of the random-effects model (Hedges, 1983).

In a meta-analysis with $k$ studies, let $i=1,\dots ,k$ index the individual studies. The random-effects model states that the observed effect sizes, denoted as ${y}_{i}$, will differ across studies due to two sources of variability. First, studies with fewer observations are more likely to yield an effect estimate ${y}_{i}$ further away from their corresponding effect parameter, denoted as ${\theta }_{i}$. The differences between ${y}_{i}$ and ${\theta }_{i}$, denoted as ${\varepsilon }_{i}$, are assumed to be normally distributed with mean equal to zero and variance ${\sigma }_{i}^{2}$, with ${\sigma }_{i}^{2}$ treated as known in practice and fixed to its observed value (Jackson & White, 2018). This first source of variability, often referred to as within-study or sampling variance, is therefore greater for those studies with a smaller sample size.

In the random-effects model, we then assume that the effect parameters ${\theta }_{i}$ of the $k$ studies included in a meta-analysis are a random sample from a population or distribution of possible effects with mean ${\mu }_{\theta }$. The differences between ${\theta }_{i}$ and ${\mu }_{\theta }$, denoted as ${u}_{i}$, are typically assumed to be normally distributed with mean equal to zero and variance ${\tau }^{2}$. This second source of variability is called between-study variance or simply heterogeneity.

When there is evidence of heterogeneity (i.e., ${\widehat{\tau }}^{2}>0$ and/or the null hypothesis ${H}_{0}:{\tau }^{2}=0$ is rejected), a natural next step is to look for moderator variables (i.e., study-level characteristics) that can explain or account for this variability. A commonly used tool for examining the influence of one or multiple moderator variables on the effect size estimates is the mixed-effects meta-regression model (Tipton et al., 2019; Viechtbauer et al., 2015). This model has the form

1 $${y}_{i} ={\beta }_{0}+{\beta }_{1}{x}_{i1}+\dots +{\beta }_{p}{x}_{ip}+{u}_{i}+{\varepsilon }_{i},$$

where ${x}_{i1}$, ..., ${x}_{ip}$ are the values of the *p* moderator variables for the ${i}^{th}$ study, coefficients ${\beta }_{1}$, ..., ${\beta }_{p}$ are the model coefficients for the moderators, quantifying the association of each moderator with the effect size magnitude, whereas ${\beta }_{0}$ represents the model intercept, while ${\varepsilon }_{i}$ and ${u}_{i}$ were defined previously (except that ${u}_{i}$ now represents residual heterogeneity not accounted for by the moderators included in the model). After fitting model (1), ${\widehat{\beta }}_{0}+{\widehat{\beta }}_{1}{x}_{i1}+\dots +{\widehat{\beta }}_{p}{x}_{ip}$ yields the predicted average effect for the combination of the moderator values present in the ${i}^{th}$ study (or one can plug in any other combination of values for the moderator variables to obtain the corresponding predicted effect).

Similar to the random-effects model, mixed-effects meta-regression models are focused on modeling the average effect size, paying less attention to the variance parameter. Indeed, in both models, ${\tau }^{2}$ is assumed to be homoscedastic. Therefore, mixed-effects meta-regression models only allow to account for the differences in ${\mu }_{\theta }$ across the moderators examined, whereas hypotheses concerning associations between the moderators and the amount of heterogeneity in the effect sizes cannot be explored. To examine the latter, we need to consider not only how ${\mu }_{\theta }$ changes, but also how ${\tau }^{2}$ changes across the levels of the moderators examined.

### Location-scale models

Location-scale models have been recently proposed as an analysis tool in the field of meta-analysis for this purpose (Viechtbauer & López-López, 2022). Location-scale models allow for modeling the two main parameters in the random-effects meta-analytic framework, namely ${\mu }_{\theta }$ and ${\tau }^{2}$, as a function of the same or different moderator variables. Here, the basis of these models is briefly introduced, but for a more detailed explanation of the estimation and inferential methods regarding the model parameters, the reader is referred to the paper by Viechtbauer and López-López (2022). In essence, the location part of the model refers to the average of the true effects, ${\mu }_{\theta }$, which is modeled as shown in Eq. (1). On the other hand, the scale part corresponds to the variance of the distribution of the true effects or heterogeneity, ${\tau }^{2}$, which can be expressed by

2 $$\text{ln}\left({\tau }_{i}^{2}\right)={\alpha }_{0}+{\alpha }_{1}{z}_{i1}+\dots +{\alpha }_{q}{z}_{iq},$$

where ${z}_{i1}$, ..., ${z}_{iq}$ are the values of the *q* moderator variables that may be related to the amount of heterogeneity, coefficients ${\alpha }_{1}$, ..., ${\alpha }_{q}$ reflect the change in $\text{ln}({\tau }_{i}^{2})$ as the moderator variables increase by one unit, and ${\alpha }_{0}$ represents the model intercept or the value of $\text{ln}({\tau }_{i}^{2})$ when all moderators are equal to zero. Note that the log link is used here to ensure that only non-negative values can be obtained for ${\tau }_{i}^{2}$ (i.e., ${\tau }_{i}^{2}$ is given by $\text{exp}\left({\alpha }_{0}+{\alpha }_{1}{z}_{i1}+\dots +{\alpha }_{q}{z}_{iq}\right)$), as would be expected for a variance component.

Location-scale models can help researchers address important questions. For instance, it is of great importance to know under what circumstances the benefits of a medical or psychological treatment are found to be stable (i.e., homogeneous) across studies (e.g., the efficacy of a treatment might vary more or less depending on the characteristics of the patients examined). Similarly, even if therapy helps prevent a disease or improve physical or mental health conditions, recovery may be more consistent or homogeneous for certain types of diagnoses than for others. Furthermore, even if there is empirical evidence for the benefits of a specific treatment, it is important to look for those conditions (or combination of moderator levels) that lead to a preferable mean outcome (according to the direction of the desired effect) and less variability (more homogeneous effects).

As an example, cognitive-behavioral therapy (CBT) has proven effective for reducing some comorbid internalizing symptoms, such as depression, anxiety, and emotional dysregulation, as well as enhancing the perceived quality of life in adults with attention-deficit or hyperactivity disorder (ADHD; López-Pinar et al., 2020). Regarding depressive symptoms, López-Pinar et al. (2020) showed that the pooled effect size (standardized mean change in between-subject studies) for CBT therapy was smaller immediately after treatment (AT; SMD = 0.27) than at the follow-up assessment (FU; SMD = 0.52), with evidence that effects immediately after treatment were more heterogeneous (${\widehat{\tau }}^{2}$ = 0.07) than at the follow-up assessment (${\widehat{\tau }}^{2}$ = 0). These authors also found that the heterogeneity found for the AT between-group effects could be explained to some extent by certain study characteristics. Among these characteristics, López-Pinar et al. (2020) found that the effect estimates were significantly larger for studies that had applied individual versus group therapy (${\chi }^{2}\left(2\right)=8.26$, $p=.02$). The use of a location-scale model would have allowed testing whether the therapy type also influences the heterogeneity of these effects. For illustrative purposes, suppose that studies in which group therapy was applied obtained not only smaller effects, but also more heterogeneous ones, than studies in which individual therapy was applied. This conclusion would strengthen the available evidence in favor of individual versus group CBT therapy to improve depressive symptoms associated with ADHD, as it shows not only higher but also more homogeneous effects.

Another case is the study of the mortality in hospitalized patients with cancer and coronavirus disease. Based on the results from a meta-regression model, Desai et al. (2021) found that the mortality rate was significantly predicted by the interaction between the patients’ mean age and the percentage of patients receiving cancer treatment at the moment of the study, as well as by the percentage of males in the sample. However, no conclusions were drawn regarding how these moderators might affect the variability of the mortality rates. For instance, assume that by using a location-scale model, we found that the interaction between age and treatment was also statistically significant for the amount of heterogeneity, for example, because within studies comprising older participants, those with a higher percentage of patients on active cancer treatment showed more variable mortality rates as well. Such a conclusion would show that the mortality rates found by those studies comprising mainly older patients undergoing cancer treatment could deviate from their mean with a greater probability, leading to both higher and lower rates than expected.

These are just examples to motivate the use of location-scale models, and an easy-to-use implementation for applying them is now available via the *metafor* package (Viechtbauer, 2010) in R (R Core Team, 2021). Within this package, different settings can be adjusted to fit location-scale models. The scale (${\alpha }_{0}$, ..., ${\alpha }_{q}$) and location (${\beta }_{0}$, ..., ${\beta }_{p}$) coefficients of a particular model can be estimated using either maximum likelihood (ML) or restricted maximum likelihood (REML) estimation (Patterson & Thompson, 1971; Patterson, 1974). Once the estimates of the scale and location coefficients are obtained, statistical inferences about these estimates can be carried out by computing Wald-type, permutation, and likelihood-ratio tests of the null hypothesis ${H}_{0}: {\alpha }_{j}=0$ (where ${\alpha }_{j}$ refers to the ${j}^{th}$ scale coefficient) and analogously for testing ${H}_{0}: {\beta }_{j}=0$. The Wald-type test (Raudenbush, 2009) is a parametric procedure that relies on the standard normal distribution as the sampling distribution of the test statistic under the null hypothesis. Conversely, the permutation test (Follmann & Proschan, 1999; Higgins & Thompson, 2004) provides a distribution-free alternative that constructs the sampling distribution of the test statistic under the null hypothesis empirically by repeatedly permuting the data. Lastly, likelihood-ratio tests (Huizenga et al., 2011) are used to compare the specified location-scale model (with one or several moderators) models with a null model (where one or multiple moderators are removed) based on their respective log-likelihoods.

Confidence intervals (CIs) around ${\widehat{\alpha}}_{j}\,\text{and}\;{\widehat{\beta}}_{j}$ can be obtained by using either Wald-type or profile-likelihood approaches. Wald-type CIs are constructed with ${\widehat{\alpha }}_{j}\pm crit\times SE[{\widehat{\alpha}}_{j}]$, where $SE[{\widehat{\alpha}}_{j}]$ is the standard error of the scale coefficient and $crit$ is an appropriate ‘critical value’ from a standard normal distribution (e.g., 1.96 for a 95% CI) and analogously for location coefficients. On the other hand, profile-likelihood CIs (Hardy & Thompson, 1996) include the set of all those values for the coefficient of interest for which the likelihood-ratio test would not be rejected given a significance level of $\alpha$ when the parameter is constrained to these values under the null model.

Although an implementation of location-scale models for meta-analysis is now available in the *metafor* package, the performance of such models – and the impact of different estimators and inference procedures – has yet to be examined by means of a comprehensive simulation study. In the present study, we focused on inferences for moderators of the amount of heterogeneity, as this is the most distinctive advantage of location-scale models over standard mixed-effects meta-regression models for which various inferential methods have already been examined in prior work (e.g., Viechtbauer et al., 2015). In this regard, we expect better performance of REML-based procedures over ML-based ones, as reported in previous simulation studies of standard random- and mixed-effects models (e.g., López-López et al., 2014; Viechtbauer, 2005). Moreover, if prior comparisons of inferential methods for location coefficients are indicative of the performance of the methods described above for making inferences about scale coefficients, the simulation results obtained by Huizenga et al. (2011) and by Viechtbauer et al. (2015) might suggest that likelihood-based methods will produce type I error rates closer to the nominal level compared to Wald-type tests. Furthermore, we also expect permutation tests to yield acceptable type I error rates based on the results obtained by Follmann and Proschan (1999) and by Viechtbauer et al. (2015). However, it is important to note that these findings were based on mixed-effects meta-regression models, and different results could be obtained in a simulation study based on location-scale models.

### Simulation methods

In the previous section, we briefly described the application of location-scale models in meta-analysis, which allow researchers to test moderators not only for the size of the average effect/outcome, but also for the amount of heterogeneity. In the present study, we aimed to examine the performance of such models, and in particular the corresponding inferential methods, when the scale part of the model includes either a qualitative (dichotomous) or continuous moderator. For this, we carried out a Monte Carlo simulation study where we simulated data using the standardized mean difference – a popular index in the social and health sciences (Fanelli et al., 2017) – as the outcome measure. In this section, the simulation factors, the data generation methods, and the performance criteria evaluated are explained and motivated.

### Data generating process

Data for the studies were generated following a two-group (experimental and control) design with a continuous dependent variable, and the outcome measure used to quantify the difference between the groups (i.e., the effect size) was the standardized mean difference, also known as Cohen’s *d* (Cohen, 1988). So far, the notation used to refer to the true and the estimated effect sizes has been ${\theta}_{i}$ and ${y}_{i}$, irrespective of the effect size index. Since from now on we will focus on the standardized mean difference, we will refer to the true and estimated effects as ${\delta}_{i}$ and ${g}_{i}$, respectively, following commonly used notation for this specific effect size measure.

To simulate a single meta-analysis, $k$ true effect sizes ${\delta}_{i}$ were generated, one for each study. In the qualitative moderator conditions, half of the $k$ true effects were randomly generated from $N({\mu}_{\delta}, {\tau}_{1}^{2})$, whereas the other half from $N({\mu}_{\delta}, {\tau}_{2}^{2})$. ${\mu}_{\delta}$ was set to 0.5, whereas ${\tau}_{1}^{2}$ and ${\tau }_{2}^{2}$ were set to the heterogeneity values assigned to the two levels of the qualitative moderator in that specific condition (to be detailed further below). In the quantitative moderator conditions, $k$ different values of ${\tau}_{i}^{2}$ were computed following the expression $\text{exp}({\alpha}_{0}+{\alpha}_{1}{z}_{i1})$, where ${z}_{i1}$ is a random value drawn from a uniform $U\left(0, 1\right)$ distribution that represents the moderator variable, and coefficients ${\alpha}_{0}$ and ${\alpha}_{1}$ were computed with ${\alpha}_{0}=\text{ln}({\tau}_{1}^{2})$ and ${\alpha}_{1}=\text{ln}({\tau}_{2}^{2})-\text{ln}({\tau}_{1}^{2})$, so that ${\tau}_{1}^{2}$ and ${\tau}_{2}^{2}$ correspond to the true amounts of heterogeneity when ${z}_{i1}=0$ and $1$, respectively. Subsequently, the true effects for the *k* studies were randomly generated from $N\left({\mu}_{\delta}, {\tau}_{i}^{2}\right)$.

Next, following the work of Hedges (1982), an observed Cohen’s *d* value for each study ${d}_{i}$ was randomly sampled from a distribution that was $1/\sqrt{{\widetilde{n}}_{i}}$ times a noncentral *t* random variable, where ${\widetilde{n}}_{i}={n}_{i}^{E}\bullet {n}_{i}^{C}/({n}_{i}^{E} + {n}_{i}^{C})$ and letting ${n}_{i}^{E}$ and ${n}_{i}^{C}$ denote the sample sizes for the experimental and control groups, respectively. The *t* distribution had a noncentrality parameter equal to $\sqrt{{\widetilde{n}}_{i}}{\delta}_{i}$ and ${m}_{i}$ degrees of freedom, where ${m}_{i}={n}_{i}^{E} + {n}_{i}^{C}-2$. Hedges (1981) showed that ${d}_{i}$ is a positively biased estimator of ${\delta}_{i}$ and derived an unbiased estimator, which is computed as ${g}_{i}=c({m}_{i})\bullet {d}_{i}$ (often referred to as Hedges’ g), where $c({m}_{i})$ is a correction factor for small sample sizes given by

3 $$c\left({m}_{i}\right)=\frac{\Gamma \left(\frac{{m}_{i}}{2}\right)}{\sqrt{\frac{{m}_{i}}{2}} \bullet \Gamma \left[\frac{\left({m}_{i}-1\right)}{2}\right]} ,$$

where $\Gamma ()$ represents the gamma function. Once the value of ${g}_{i}$ was obtained, an unbiased estimate of its sampling (or within-study) variance was computed with (Hedges, 1983)

4 $${\widehat{\sigma}}_{i}^{2}\left({g}_{i}\right)={\frac{1}{\widetilde{n}}}_{i}+\left[1-\frac{1}{\left(\frac{{c\left({m}_{i}\right)}^{2} \bullet {m}_{i}}{{m}_{i}-2}\right)}\right]{\left({g}_{i}\right)}^{2},$$

### Simulation conditions

The factors manipulated in this simulation were the number and sample size of the studies, the type of moderator variable, and the amount of heterogeneity across the different levels/categories of the moderator variable. To identify a range of realistic scenarios in the social and health sciences, the manipulated conditions in the current study were set according to the results of a systematic review of 54 meta-analyses on the efficacy of psychological interventions using different types of effect size indices based on mean differences (Rubio-Aparicio et al., 2018).

For the number of studies, $k$, ten values were considered, namely 10 to 100 in steps of ten, corresponding to a small to large number of studies for the meta-analysis. To model the sample sizes of the studies, $N$, we followed the sample size distributions of those 54 meta-analyses included by Rubio-Aparicio et al. (2018). Those distributions were positively skewed, with an average skewness coefficient of 1.423. To emulate similar distributions, we set five fixed values that were repeated within the same meta-analysis (until a sample size was assigned to each study), representing a skewed distribution of total samples sizes with average values of 30, 50, and 100. Specifically, values {12, 16, 18, 20, 84} were set for a sample size distribution with mean equal to 30, values {32, 36, 38, 40, 104} for a distribution with mean equal to 50, and values {82, 86, 88, 90, 154} for a distribution with mean equal to 100. The size of the experimental and control groups was assumed to be the same (e.g., $N/2$).

As described above, two types of moderators were simulated, namely a qualitative (dichotomous) moderator and a quantitative moderator. Furthermore, a wide range of values for the between-studies variance or heterogeneity ${\tau }^{2}$ was considered, namely, 0.01, 0.02, 0.04, 0.08, 0.16, 0.32, and 0.64. Each of these ${\tau}^{2}$ values were combined with themselves and with each other, resulting in a total of 28 comparisons. The ${\alpha}_{1}$ values resulting from these 28 comparisons tend to repeat, as the ${\tau}_{2}^{2}$/${\tau}_{1}^{2}$ comparisons always result in a ratio of 1, 2, 4, 8, 16, 32, and 64, and can be found in the supplemental materials available at the Open Science Framework (Blázquez-Rincón et al., 2022; osf.io/ax27z). These values represent a constant growth of the ratio between one ${\tau }^{2}$ value and the next and were set with the goal of considering two different aspects. On the one hand, we were interested in the effect of the difference in the amount of heterogeneity for the groups defined by the moderator variable; for instance, the two levels defined by the qualitative moderator can be assigned to ${\tau }^{2}$ values of 0.01 and 0.02, 0.01 and 0.04, 0.01 and 0.08, and so on, which implies an increasing difference in the amount of heterogeneity between the two levels. On the other hand, for a constant difference, we were interested in how changing the amounts of heterogeneity assigned to these levels might affect the performance of the model. For example, when the ${\tau}^{2}$ values for the two levels were set to 0.01 and 0.02 or 0.08 and 0.16, then in both cases the ratio ${\tau}_{2}^{2}/{\tau}_{1}^{2}$ is the same, but the overall amount of heterogeneity is larger in the latter case.

Therefore, in total we examined 1680 conditions (2 types of moderators × 10 values of $k$ × 3 sample sizes × 28 pairs of ${\tau}^{2}$ values). For each condition, 10,000 meta-analyses were simulated (except when examining the performance of the permutation test; see below).

### Analytic procedures

The simulation was programmed in R 4.1.0 (R Core Team, 2021) using the *metafor* package (v3.1.18; Viechtbauer, 2010) for the model fitting and carrying out the inferential methods examined. The R code used for the simulation and analyses reported below are available at the Open Science Framework (Blázquez-Rincón et al., 2022; osf.io/ax27z).

In each meta-analysis, both the specified location-scale model (with a moderator variable for the scale part) and the null model (without the moderator variable) were fitted using ML and REML estimation. From the specified location-scale model, the three significance tests (Wald-type, permutation, and likelihood-ratio tests) and both CIs (Wald-type and profile-likelihood) were then obtained regarding the scale coefficient ${\alpha}_{1}$. We used a significance level of $\alpha =0.05$ for the tests and, correspondingly, constructed 95% confidence intervals.

### Constraints on the coefficient estimates

It is important to note that convergence problems may arise when fitting location-scale models since several of the involved procedures require iteration (the estimation of the location and scale coefficients, the numerical approximation of the Hessian matrix, and the computation of profile-likelihood CIs). Apart from that, numerical issues may also arise when estimating the scale coefficients. Following Eq. (2), scale coefficients are defined by the natural logarithm of ${\tau}_{i}^{2}$. For example, when we fit a location-scale model with a dichotomous moderator for the scale part, the scale part of the model would be represented as $\text{ln}({\tau}_{i}^{2})={\alpha}_{0}+{\alpha}_{1}{z}_{i1}$, where ${z}_{i1}$ is set to zero (for the first moderator level) or to one (for the second level). Therefore, ${\alpha}_{0}=\text{ln}({\tau}_{1}^{2})$, while ${\alpha }_{1}=\text{ln}\left({\tau}_{2}^{2}\right)-\text{ln}({\tau}_{1}^{2})$, with ${\tau }_{1}^{2}$ and ${\tau }_{2}^{2}$ denoting the ${\tau }^{2}$ values for the first and second moderator levels, respectively. If ${\tau }_{1}^{2}=0$, then ${\alpha}_{0}=-\infty$ and ${\alpha}_{1}=\infty$ as long as ${\tau}_{2}^{2}>0$ (to compensate for ${\alpha}_{0}$). When fitting the model under such a scenario, the parameter estimates may therefore try to drift towards these extremes (a phenomenon similar to what can occur in logistic regression under perfect separation). This will affect not only the point estimates but also the standard errors of ${\widehat{\alpha}}_{0}$ and ${\widehat{\alpha}}_{1}$ (which either may become extremely large or cannot be computed at all based on the numerical approximation to the Hessian matrix that is used). This may adversely affect Wald-type tests and CIs. In addition, profile-likelihood CIs could also be affected by the space of possible ${\widehat{\alpha}}_{0}$ and ${\widehat{\alpha}}_{1}$ values that needs to be examined for their construction.

Given the numerical problems that may arise, we applied some constraints regarding the space of possible ${\widehat{\alpha}}_{0}$ and ${\widehat{\alpha}}_{1}$ values when fitting the specified models. The space of ${\widehat{\alpha}}_{0}$ and ${\widehat{\alpha}}_{1}$ values examined is related to the maximum and minimum ${\widehat{\tau }}^{2}$ values that the researcher expects to find in all the groups defined by the moderator variable, as explained previously. Therefore, as ${\tau}^{2}$ values ranged from 0.01 to 0.64 in the present simulation, actual ${\alpha}_{0}$ values ranged from – 4.61 to – 0.45, whereas values for ${\alpha}_{1}$ ranged from 0 to 4.15. Consequently, the space of possible values for ${\widehat{\alpha}}_{0}$ was constrained to [– 10, 1], whereas for ${\widehat{\alpha}}_{1}$ was constrained to [– 11, 11].^1^ This way, extreme values for the estimates of both scale coefficients were avoided, but by using these wide intervals, no unrealistic knowledge of the possible size of the coefficients was introduced. In addition, we also set the lower and the upper bounds of the Wald-type confidence interval for ${\widehat{\alpha}}_{1}$ to [– 11, 11] in case the obtained bounds were more extreme, in order to make a fairer comparison between the Wald-type and the profile-likelihood confidence intervals.

### Performance criteria

To compare the alternatives described above for fitting and making inferences in the context of location-scale models, we focused on the following criteria. First, we examined the rejection rates of the Wald-type, permutation, and likelihood-ratio tests regarding ${\widehat{\alpha}}_{1}$ across the simulation conditions. The rejection rates for each test were computed as the proportion of the number of meta-analyses for which the test yielded a *p* value under 0.05 out of the total number of meta-analyses simulated within a single condition. Our goal was to determine which of these tests controls the type I error rate (i.e., keeping the rejection rate when the null hypothesis ${H}_{0}:{\alpha}_{1}=0$ is true at or below the significance level of 0.05) across the various conditions and, among those that do control the type I error rate, which test shows the highest statistical power (i.e., the highest rejection rate when the null hypothesis is false).

Concerning the Wald-type and the profile-likelihood confidence intervals for ${\alpha}_{1}$*,* we computed their coverage probabilities as the proportion of simulated meta-analyses where the confidence interval obtained included the true value set for ${\alpha}_{1}$. Furthermore, the mean width of both confidence intervals was also examined across the simulation conditions. Here, our goal was to determine which method for constructing confidence intervals for ${\alpha}_{1}$ keeps the coverage probabilities closest to the nominal 0.95 level across the various conditions. Should the coverage probabilities be similarly close to the nominal level for the two methods, then the method that provides the narrower intervals will be considered the best. Note that which test and method for constructing confidence intervals is to be preferred might also depend on the estimation method used (ML versus REML).

Although these four criteria were our primary interest and the only ones discussed in the Results section, we additionally computed the absolute bias in the predicted values of ${\tau}_{1}^{2}$ and ${\tau}_{2}^{2}$ based on the fitted model, and the absolute bias and mean squared error of the estimates of the scale coefficients ${\alpha}_{0}$ and ${\alpha}_{1}$. Results with respect to these variables can be found in the supplemental materials.

Finally, even with the constraints imposed on the space of the scale coefficients described earlier, convergence or numerical issues can still arise. This may pertain to the model fitting itself (either with respect to the location-scale model or the null model, which is needed for conducting the likelihood-ratio test) but can also affect the computation of the Hessian matrix (needed for computing the Wald-type test and confidence interval, as well as the permutation test) or the construction of the profile-likelihood confidence intervals. If a convergence or numerical issue arose in any of these steps, an additional iteration was run, so that all results obtained were based on at least the intended number of simulated meta-analyses in each condition. Moreover, the proportion of iterations within each simulation condition in which any of these issues arose (i.e., problems with fitting of the (null) model, computation of the Hessian matrix, or construction of the profile-likelihood confidence interval) were saved and will be discussed alongside the rest of the performance criteria.

It is important to mention that the permutation test is a computationally intensive procedure since it requires fitting the same model multiple times, ideally for all possible permutations. This quickly becomes infeasible (e.g., for $k=10$, there are already $10!=\text{3,628,800}$ possible permutations of the moderator values). Instead, we used 1000 random permutations, which should provide a reasonably accurate approximation to the exact permutation-based *p* value^2^. However, the time and computational resources required to perform the permutation test on 10,000 meta-analyses per simulation condition were too high. For this reason, we decided to perform the permutation test only on 1000 meta-analyses per condition, while the other procedures were performed on at least 10,000 meta-analyses per condition. This way of proceeding does not affect the results (it just implies that the type I error rate and power of the permutation test are estimated with less accuracy), while allowing us to examine all methods in a reasonable time considering the available resources. Even with this restriction, the computational demands on this simulation study were substantial. Therefore, the simulation was run on a cluster computer using 600 cores (AMD EPYC 7551 CPUs), requiring approximately 6 days for completion (approximately 83,100 core hours).

## Simulation results

### Type I error rates and statistical power

**Fig. 1:**
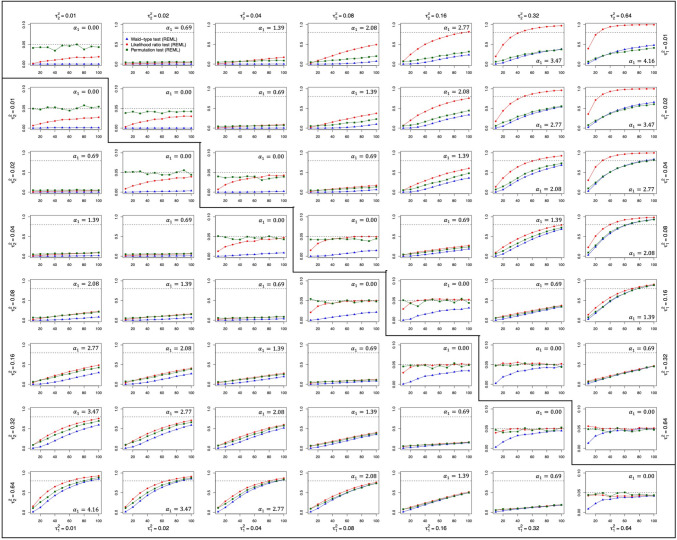
Rejection rate of the significance tests for parameter α_1_. *Note.* The upper triangular part of the matrix contains the data for the qualitative moderator, whereas the lower triangular part contains the data for the quantitative moderator. In both cases, the y-axis represents the rejection rate of the significance tests for parameter a 1 and the x-axis refers to the number of studies within a single meta-analysis

**Fig. 2:**
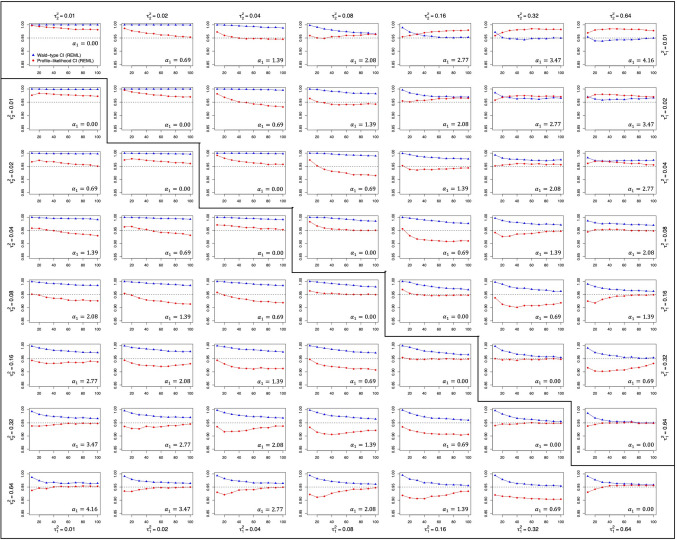
Coverage probability of the confidence intervals for parameter α_1_. *Note.* The upper triangular part of the matrix contains the data for the qualitative moderator, whereas the lower triangular part contains the data for the quantitative moderator. In both cases, the y-axis represents the coverage probability of the confidence intervals for parameter a1 and the x-axis refers to the number of studies within a single meta-analysis

**Fig. 3:**
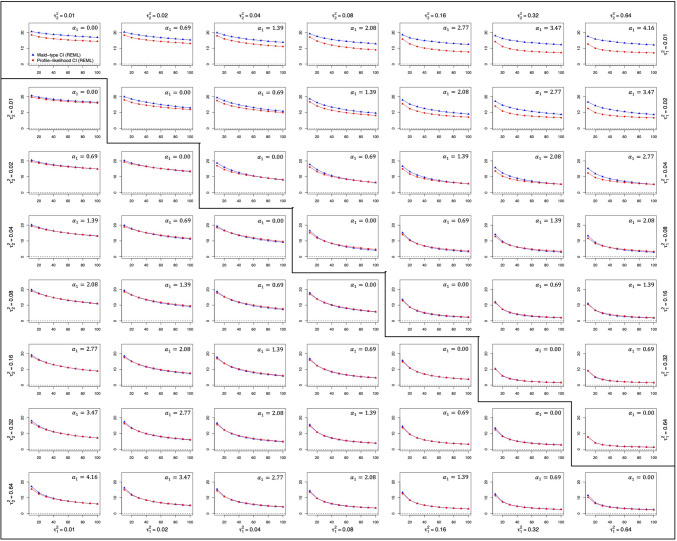
Width of the confidence intervals for parameter α_1_. *Note.* The upper triangular part of the matrix contains the data for the qualitative moderator, whereas the lower triangular part contains the data for the quantitative moderator. In both cases, the y-axis represents the width of the confidence intervals for parameter a1 and the x-axis refers to the number of studies within a single meta-analysis

**Fig. 4:**
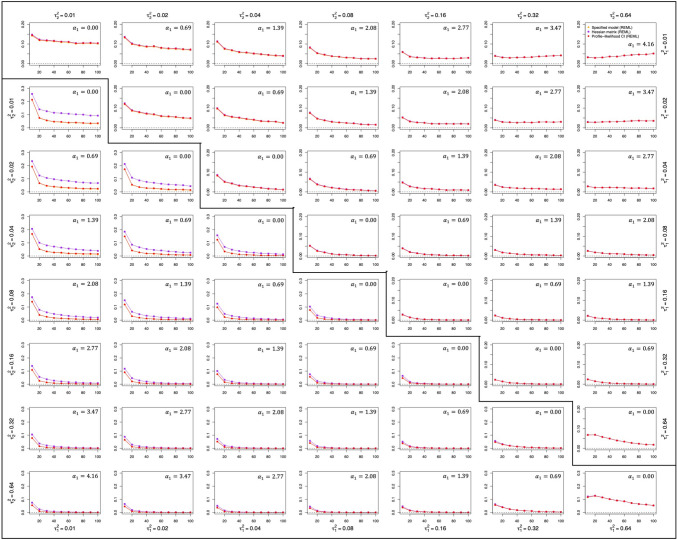
Non-convergence rates for the specified models, hessian matrix, and profile-likelihood confidence intervals. *Note.* The upper triangular part of the matrix contains the data for the qualitative moderator, whereas the lower triangular part contains the data for the quantitative moderator. In both cases, the y-axis represents the nonconvergence rates and the x-axis refers to the number of studies within a single meta-analysis

Regarding the significance tests for ${\widehat{\alpha }}_{1}$, we report type I error rates given a significance level of 0.05 whenever the difference between the ${\tau}^{2}$ values for the levels of the moderator was zero (i.e., for ${\alpha}_{1}=0$, or equivalently, when the ${\tau}_{2}^{2}/{\tau}_{1}^{2}$ ratio was 1), and statistical power in those scenarios where the ${\tau}^{2}$ values were different (i.e., for ${\tau}_{2}^{2}/{\tau}_{1}^{2}$ ratios larger than 1). Figure 1 depicts the rejection rates of the Wald-type, likelihood-ratio, and permutation tests according to the number of studies within a single meta-analysis. Figure 1 includes the results when REML estimation was used, while Figure S1 of the supplemental materials presents the results for ML estimation (and analogously for Figs. 2, 3 and 4, to be discussed further below). In all figures, the upper triangular part of the matrix shows the results for the qualitative moderator, while the lower triangular part contains the results for the quantitative moderator. The average sample size of the studies did not affect the results of any outcome variable substantially and, therefore, conditions with different sample sizes were averaged when creating the plots.

Overall, all significance tests showed higher statistical power and type I error rates closer to the significance level the greater the number of studies and the higher the ${\tau }^{2}$ values set for the levels of the moderator. In addition, all significance tests obtained slightly higher rejection rates when the estimation method used was ML rather than REML, which sometimes led to inflated type I error rates.

The permutation test obtained type I error rates closer to the nominal level of 0.05 than the Wald-type and likelihood-ratio tests, regardless of the number of studies and the amount of heterogeneity set for the levels of the moderator. On the other hand, in the case of the likelihood-ratio test, the ${\tau }^{2}$ values had to be greater than 0.08 to get reasonable type I error rates with at least 30 studies. Furthermore, the type I error rates for the likelihood-ratio test exceeded the nominal rate of 0.05 when the ${\tau}^{2}$ values were greater than 0.16 and ML estimation was used while, on the contrary, they remained close to 0.05 for REML estimation even if the ${\tau}^{2}$ values were 0.16 or greater. The Wald-type test was the procedure that showed the most conservative type I error rates, yielding results below the nominal level in most scenarios. This significance test only performed close to the nominal level for scenarios with more than 80 studies and ${\tau}^{2}$ values of 0.32 or higher.

Regarding statistical power, the likelihood-ratio test obtained higher rejection rates, especially in scenarios where a qualitative moderator (rather than a quantitative moderator) was examined. In contrast, the Wald-type and the permutation tests often showed similar performance for both types of moderator variables (with occasionally a slight advantage for the permutation test). Interestingly, the statistical power of all tests was higher as the overall magnitude of the ${\tau}^{2}$ values increased, which can be easily seen by looking at the diagonals of the matrix in Fig. 1. Not surprisingly, ratios between the ${\tau}^{2}$ values further away from 1 led to higher statistical power for all significance tests, as can be seen by looking individually at the rows of the upper triangular part and the columns of the lower triangular part in Fig. 1. A power of at least 0.80 is often recommended in psychology (Cohen, 1988) and in our simulation, this was achieved in some scenarios, especially when the value of ${\tau}_{1}^{2}$, the ${\tau}_{2}^{2}/{\tau }_{1}^{2}$ ratio, and the number of studies were large. For example, for a qualitative moderator, the likelihood-ratio test obtained power close to 0.80 for ${\tau}_{1}^{2}=0.01$ and ${\tau}_{2}^{2}=0.16$ (ratio equal to 16) with around 80 studies, while the same significance test needed, on average, ten more studies to yield a similar statistical power when ${\tau}_{1}^{2}=0.02$ and ${\tau}_{2}^{2}=0.16$ (ratio equal to 8). The same occurred in the case of a quantitative moderator, except that in these scenarios larger ${\tau }_{2}^{2}/{\tau }_{1}^{2}$ ratios (i.e., bigger differences between ${\tau}_{1}^{2}$ and ${\tau}_{2}^{2}$) and a greater number of studies were needed to achieve a power of 0.80 or higher.

### Coverage probability and width

The coverage probabilities of the Wald-type and the profile-likelihood confidence intervals for ${\alpha}_{1}$ are depicted in Fig. 2, while Fig. 3 shows the corresponding mean width of both procedures. Both figures present results according to the number of studies within a single meta-analysis. Note that the Wald-type and the profile-likelihood intervals were constrained to the limits imposed on the scale coefficient ${\alpha}_{1}$ and, consequently, the width of both intervals was affected by this imposition (the maximum possible width for both intervals was 22 points, given that the limits for the estimation of ${\alpha}_{1}$ were [– 11, 11]).

Concerning the estimation method, both procedures showed slightly wider confidence intervals when ML estimation was used rather than REML, while the coverage probabilities were very similar regardless of the estimation method used.

Irrespective of the type of moderator variable, the width of both procedures tended to decrease as the number of studies increased (as can be seen within each panel within Fig. 3), as the overall magnitude of the ${\tau}^{2}$ values increased (as can be seen by looking at the diagonals of Fig. 3), and also, as the ratio ${\tau}_{2}^{2}/{\tau}_{1}^{2}$ deviated more from 1 (as can be seen by looking at the rows/columns within Fig. 3). This decrease in the interval width was also accompanied by coverage probabilities closer to the nominal level of 0.95 in the case of the Wald-type interval, that is, the Wald-type interval yielded coverage probabilities closer to the nominal level as the number of studies, the overall amount of heterogeneity, and the ${\tau}_{1}^{2}/{\tau}_{2}^{2}$ ratio increased. On the other hand, the performance of the profile-likelihood interval was not as straightforward, as increases in these characteristics could lead to coverages that were either somewhat closer or somewhat further away from the nominal level.

With respect to the type of moderator, while for the quantitative moderator both intervals performed similarly, differences were found between the width of the Wald-type and the profile-likelihood intervals in the case of the qualitative moderator. Specifically, when ${\tau}_{1}^{2}$ was small (0.02 or below), the profile-likelihood confidence interval always showed narrower intervals than the Wald-type procedure. In fact, this difference between intervals was bigger the farther apart the values of ${\tau}_{1}^{2}$ and ${\tau}_{2}^{2}$ were. In addition, slightly higher coverage probabilities were found when a qualitative rather than a quantitative moderator was analyzed. Although in general terms the profile-likelihood interval showed coverage probabilities closer to the nominal 0.95 level and the Wald-type interval tended to yield coverage probabilities above it, the performance of the profile-likelihood interval differed with respect to the type of moderator variable analyzed. While for the quantitative moderator, the profile-likelihood interval always yielded lower coverage probabilities than the Wald-type interval, in conditions with a qualitative moderator the profile-likelihood interval showed lower coverage probabilities in most scenarios but obtained greater coverage probabilities when ${\tau}_{1}^{2}$ was very small (0.02 or below) and ${\tau}_{2}^{2}$ was very high (0.32 or above). In the most extreme scenario (${\tau}_{1}^{2}=0.01$ and ${\tau}_{2}^{2}=0.64$), the usual performance of both confidence intervals was reversed: Interestingly, while the profile-likelihood procedure yielded narrower intervals than the Wald-type method, the Wald-type interval obtained coverage probabilities near 0.95, and the profile-likelihood interval yielded results near 1.

### Non-convergence rates

Figure 4 shows the proportion of iterations in which the default algorithm for fitting the specified location-scale models did not converge, the Hessian matrix could not be computed, and the profile-likelihood interval could not be constructed. For brevity, we will refer to these proportions as non-convergence rates. Note that the Hessian and profile-likelihood intervals are automatically missing when the model did not converge, and hence the non-convergence rate of the model itself is a lower bound for the rates of the latter two by construction.

The main factor that seemed to affect these rates was the number of studies (in general, non-convergence rates decreased as the number of studies increased) followed by the type of moderator and the overall amount of heterogeneity (non-convergence rates decreased with higher amounts). In general, the construction of the Hessian matrix showed the highest non-convergence rates. Indeed, this was most noticeable in the case of the quantitative moderator, whereas for the qualitative moderator, all procedures examined obtained more similar non-convergence rates. The non-convergence rates can be especially high (above 0.30) for the Hessian matrix when $k=10$ and when examining a quantitative moderator. However, as the number of studies increased (20 or more), the differences in the non-convergence rates between the qualitative and the quantitative moderators decreased. In addition, procedures based on ML estimation showed higher rates than procedures that used REML, although this difference tended to disappear as the number of studies increased.

Non-convergence rates were also related to the ${\tau}_{1}^{2}$ and ${\tau}_{2}^{2}$ values. Larger ${\tau}_{1}^{2}$ and ${\tau}_{2}^{2}$ values led to lower rates for all the procedures examined, regardless of the type of moderator variable, the number of studies, or the estimation procedure. However, this trend did not hold in the scenario in which ${\tau}_{1}^{2}$ and ${\tau}_{2}^{2}$ were equal to 0.64. In this condition, the non-convergence rates increased again for all procedures, reaching 0.13 for a quantitative moderator and 0.07 for a qualitative one when the number of studies was small (20 or below).

Finally, it is worth noting that although the general trend found was a decrease in the non-convergence rates as the number of studies included in the meta-analysis increased, this was not the case in some of the simulated scenarios. In particular, in conditions with a qualitative moderator and where the ratio between ${\tau}_{2}^{2}$ and ${\tau}_{1}^{2}$ was very large, non-convergence rates increased slightly as the number of studies increased.

## Discussion

Location-scale models are an interesting development in the field of meta-analysis since they allow researchers to examine the influence of moderator variables on the mean and variance of the distribution of true effects in a single modeling framework. In the present work, we examined the performance of various methods for drawing inferences (i.e., hypothesis tests and confidence intervals) in the context of this model. Since the main advantage of these models is the modeling of the heterogeneity parameter, we focused on the scale part of the model and examined Wald-type, permutation, and likelihood-ratio tests and Wald-type and profile-likelihood intervals for the coefficient relating either a dichotomous or a quantitative moderator variable to the amount of heterogeneity in the effect sizes.

Concerning the significance rates, the permutation test showed more desirable type I error rates than the Wald-type and likelihood-ratio tests. Although the type I error rates of the latter two types of tests approached those of the permutation test as the number of studies and the amount of heterogeneity increased, the permutation test rates were near the nominal level of 0.05 regardless of these simulation factors. This result mirrors the findings from Viechtbauer et al. (2015) on methods for testing location coefficients, where the permutation test also showed better control of the type I error rate than the other two types of tests. This finding is not entirely unexpected, given that the permutation test is, in principle, an exact test. Therefore, the permutation test was expected to maintain the type I error rate at the desired significance level of the test. On the other hand, the Wald-type and likelihood-ratio tests rely on asymptotics, and as expected, these tests obtained type I error rates closer to the nominal level as the number of studies increased. With respect to the ${\tau}^{2}$ values set for the levels of the moderator variable, it is notable that the type I error rates of the likelihood-ratio test, and especially those of the Wald-type test, approached the nominal level of 0.05 as the overall amount of heterogeneity increased. Furthermore, the significance rates of all tests did not greatly depend on the estimation method used (ML or REML). However, for ${\tau}^{2}$ values of 0.32 or greater, the type I error rates obtained with the likelihood-ratio test based on ML estimation were inflated for meta-analyses with fewer than 50 studies.

Regarding statistical power, the likelihood-ratio test obtained higher rejection rates, especially in those scenarios where a qualitative moderator (rather than a quantitative moderator) was examined. In contrast, the Wald-type and the permutation tests showed similar and inferior performance for both types of moderator variables. Still, it is important to be cautious about the statistical power of the likelihood-ratio test, given that it only achieved an acceptable statistical power (close to 0.80) in scenarios with a high number of studies and a considerable difference between ${\tau}_{1}^{2}$ and ${\tau}_{2}^{2}$. Interestingly, not only was the statistical power of the significance tests higher the greater the difference between the ${\tau}^{2}$ values set for the levels of the moderator variable (which was to be expected), but also as the overall amount of heterogeneity increased (i.e., while keeping the ratio of ${\tau}_{1}^{2}$ and ${\tau}_{2}^{2}$ constant). In other words, location-scale models are more likely to detect relevant moderators of the amount of heterogeneity when there is a large amount of heterogeneity to begin with.

With respect to the methods for constructing confidence intervals around the scale coefficient, result patterns are less straightforward. When ${\tau }_{1}^{2}={\tau }_{2}^{2}$, the profile-likelihood interval performed best, as it showed lower coverage probabilities than the Wald-type interval in most scenarios but closer to the nominal 95% level, and slightly narrower intervals. In these cases, the REML estimation method yielded more desirable results than the ML procedure. However, these trends varied depending on the type of moderator variable examined when ${\tau }_{1}^{2}\ne {\tau }_{2}^{2}$. When a quantitative moderator was under study, the profile-likelihood interval always showed lower coverage probabilities than the Wald-type interval. The greater the ${\tau}_{1}^{2}$ and ${\tau}_{2}^{2}$ values (keeping the ${\tau}_{1}^{2}/{\tau}_{2}^{2}$ ratio constant), the lower the coverage probabilities obtained by both procedures. On the contrary, when a qualitative moderator was analyzed, there were scenarios where the profile-likelihood interval obtained lower coverage probabilities than the Wald-type interval and vice versa. In general, the profile-likelihood interval tended to obtain lower coverage probabilities. Nevertheless, as the difference between ${\tau}_{1}^{2}$ and ${\tau}_{2}^{2}$ increased, the Wald-type interval showed coverage probabilities closer to the nominal 95% level while the profile-likelihood procedure obtained results close to 100%, while at the same time yielding narrower intervals on average.

This phenomenon became especially pronounced when ${\tau}_{1}^{2}$ was small and ${\tau}_{2}^{2}$ increased. Inspection of the results of individual simulation iterations under such scenarios indicates that this appears to be a consequence of the estimate of ${\alpha}_{0}$ sometimes drifting towards its lower bound (which was set to −10) while the estimate of ${\alpha}_{1}$ then needs to compensate for this by drifting towards its upper bound (which was set to 11). As a result, the standard errors of the coefficients then become very large, leading to Wald-type confidence intervals for ${\alpha}_{1}$ which span the entire permissible range for this coefficient (i.e., – 11 to 11). When this happens, then this has a relatively minor impact on the interval coverage (obviously, the interval then covers ${\alpha}_{1}$ for this particular iteration), but a much more detrimental impact on the interval width. In fact, without imposing constraints on the parameter estimates, the width of the Wald-type confidence interval could then easily drift into the tens of thousands. In contrast, the profile-likelihood confidence interval, which does not make use of the standard errors of the coefficients, does not yield such extreme widths under such scenarios while at the same time having a coverage rate that is somewhat too high. Interestingly, problems with extreme standard errors did not appear to be an issue when the moderator variable was quantitative (at least in iterations where non-convergence issues did not arise) and hence the mean interval widths of the Wald-type and profile-likelihood intervals were then essentially identical.

## Recommendations

Although no procedure outperformed the others in all the examined scenarios, the following recommendations are suggested based on the results presented above. When the number of studies and the overall amount of heterogeneity is low, the power of all tests is so low as to make the use of location-scale models typically a fruitless endeavor to begin with. On the other hand, under more favorable circumstances (large $k$ and overall heterogeneity), the likelihood-ratio test would be our overall recommendation since it tends to outperform the other two tests in terms of power while showing adequate control of the type I error rate.

Along with the likelihood-ratio test, we also recommend using the REML-based profile-likelihood confidence interval for consistency. While the results with respect to Wald-type versus profile-likelihood confidence intervals were more mixed, and the latter could have coverage rates that dip below the nominal 95% coverage rate under some scenarios, the coverage rate was always at least 90% and hence not egregiously far away from its target.

The expected statistical power (and, also, the coverage probability) seems to be a function of the lowest heterogeneity value or ${\tau}_{1}^{2}$, the ratio ${\tau}_{2}^{2}/{\tau}_{1}^{2}$, the type of moderator analyzed, and the number of primary studies: power will be higher when a qualitative moderator variable is analyzed and as these other variables increased. Since none of these circumstances can be changed in a meta-analysis, we encourage researchers to consult the power and coverage probabilities of the potential analysis in the graphs provided in this paper based on the characteristics of their research. Only in this way will meta-analysts be able to decide, with a particular significance level in mind, whether it is appropriate to fit a location-scale model.

### Statistical constraints and non-convergence problems

As explained in the Methods section, our results are conditioned on some constraints imposed on the space of possible values for ${\widehat{\alpha}}_{0}$ and ${\widehat{\alpha}}_{1}$ with the goal of avoiding certain numerical issues. These numerical problems are more likely when the heterogeneity present in one of the moderator levels is very low. In essence, to compensate for the extremely negative value of one scale coefficient, the estimation process will lead to very high values for the other coefficient. These extreme values may affect the standard errors of ${\widehat{\alpha}}_{0}$ and ${\widehat{\alpha}}_{1}$, increasing the probability that the Hessian matrix cannot be constructed, along with the Wald-type test and confidence interval. In those cases where the Hessian matrix can be computed, the standard errors of ${\widehat{\alpha}}_{0}$ and ${\widehat{\alpha}}_{1}$ could be so large that the rejection rates of the Wald-type test become too low, and the coverage probability of the Wald-type interval reaches 100%. The lack of constraints may also affect the permutation and likelihood-ratio tests and the profile-likelihood confidence interval, leading to lower rejection rates and overly wide confidence intervals.

Imposing constraints on the space of possible values for the scale parameters was a necessary step to pursue the objectives set out in our simulation work but becomes unnecessary in applied meta-analyses. In scenarios where estimates of scale coefficients drift towards $\pm \infty$, the results obtained will not be useful in any case and imposing restrictions on the range of values for the coefficients will not help to recover meaningful results. Note that it is more likely that any of these processes will not converge when the meta-analysis includes a small number of studies, a quantitative moderator is examined, and ML estimation is used. However, it should also be noted that non-convergence issues can typically be resolved in individual cases by making adjustments to the numerical procedures used and/or by switching to a different optimizer, but automating the use of such remedial steps is very difficult in the context of a simulation study. Therefore, the present results simply indicate how often such corrective steps must be taken in practice.

## Limitations and future research

Given the many simulation conditions, factors that could have important implications for the results have been omitted. To our knowledge, this is the first simulation study that has assessed the performance of procedures for fitting location-scale models in meta-analysis. Therefore, we focused on the case where only one moderator variable is included in the scale part of the model when, in fact, it is also possible to include several moderator variables as predictors for both the scale and location parts of the model in real scenarios. For this same reason, we only examined scenarios in which the sample sizes of the groups defined by the moderator variable were balanced or where the quantitative moderator was uniformly distributed. It is uncertain whether the significance tests or confidence intervals would perform similarly to the results of the present simulation in those contexts where several moderators were included (qualitative, quantitative, or a combination thereof), as well as when group sizes are unbalanced or when moderators arise from a different distribution. Future simulation work would be necessary to achieve a deeper understanding in this regard.

Likewise, in our simulation study, we generated independent observation units; that is, it has been assumed that the effect sizes within the same meta-analysis are computed based on different samples and that no other form of hierarchical clustering or dependence (e.g., spatial or temporal) underlies the true effects. This is not what happens in some real-world scenarios where several effect sizes may have been calculated based on the same sample of subjects but using different measurement tools or where several studies included in the meta-analysis may have been performed by the same author or research laboratory. Analyses in such cases involve the use of multivariate/multilevel models, which are beyond the scope of the present manuscript.

In either case, it should be borne in mind that although location-scale models have been used previously in other research areas, their extrapolation to the field of meta-analysis implies the development and improvement of their implementation based on the special characteristics of this type of application.

## Conclusion

Our results suggest that location-scale models are a useful tool for modeling the heterogeneity parameter as a function of different types of moderating variables. Fitting these models is computationally more demanding than standard mixed-effects meta-regression models, and hence the decision to use them in practice should be made taking into account the characteristics of the meta-analytic database. Although future methodological and applied research studies are likely to provide valuable insights on their performance and capabilities, location-scale models are a powerful tool allowing researchers from a wide variety of scientific fields to address questions that could not be explored before.

## Supplementary information

Below is the link to the electronic supplementary material.

Supplementary file1 (JPG 1094 KB)

Supplementary file2 (JPG 1079 KB)

Supplementary file3 (JPG 966 KB)

Supplementary file4 (JPG 960 KB)

Supplementary file5 (R 15 KB)

## Data Availability

The datasets generated and analyzed during the current study are available in the Open Science Framework repository: 10.17605/OSF.IO/AX27Z

## Footnotes

1 Such constraints are easily to apply via the model fitting procedures in the *metafor* package. In the present case, we used rma(estimate, sampling_variance, scale = ~ zi, method="ML", control = list(alpha.min = c(– 10, – 11), alpha.max = c(1, 11)) (or method="REML") to fit the location-scale model with these constraints.

2 The actual number of permutations required to obtain the exact permutation-based *p* value can be lower when there are repeated values of the moderator. In fact, when $k=10$, there are only 252 unique permutations of the moderator values when it is dichotomous and split evenly between the two groups. In this special case, the exact *p* value was obtained. However, for larger values of $k$, the number of unique permutations again grows so large as to make this infeasible for the simulation study and hence 1000 random permutations were again used.

## Code availability

The code for the present simulation work is available in the Open Science Framework repository: 10.17605/OSF.IO/AX27Z
