# Supplementary figures and images for: Performance of location-scale models in meta-analysis: A simulation study
Source: Behav Res Methods. 2025 Mar 17;57(4):118. doi: 10.3758/s13428-025-02622-5 (PMC11914364; doi:10.3758/s13428-025-02622-5)

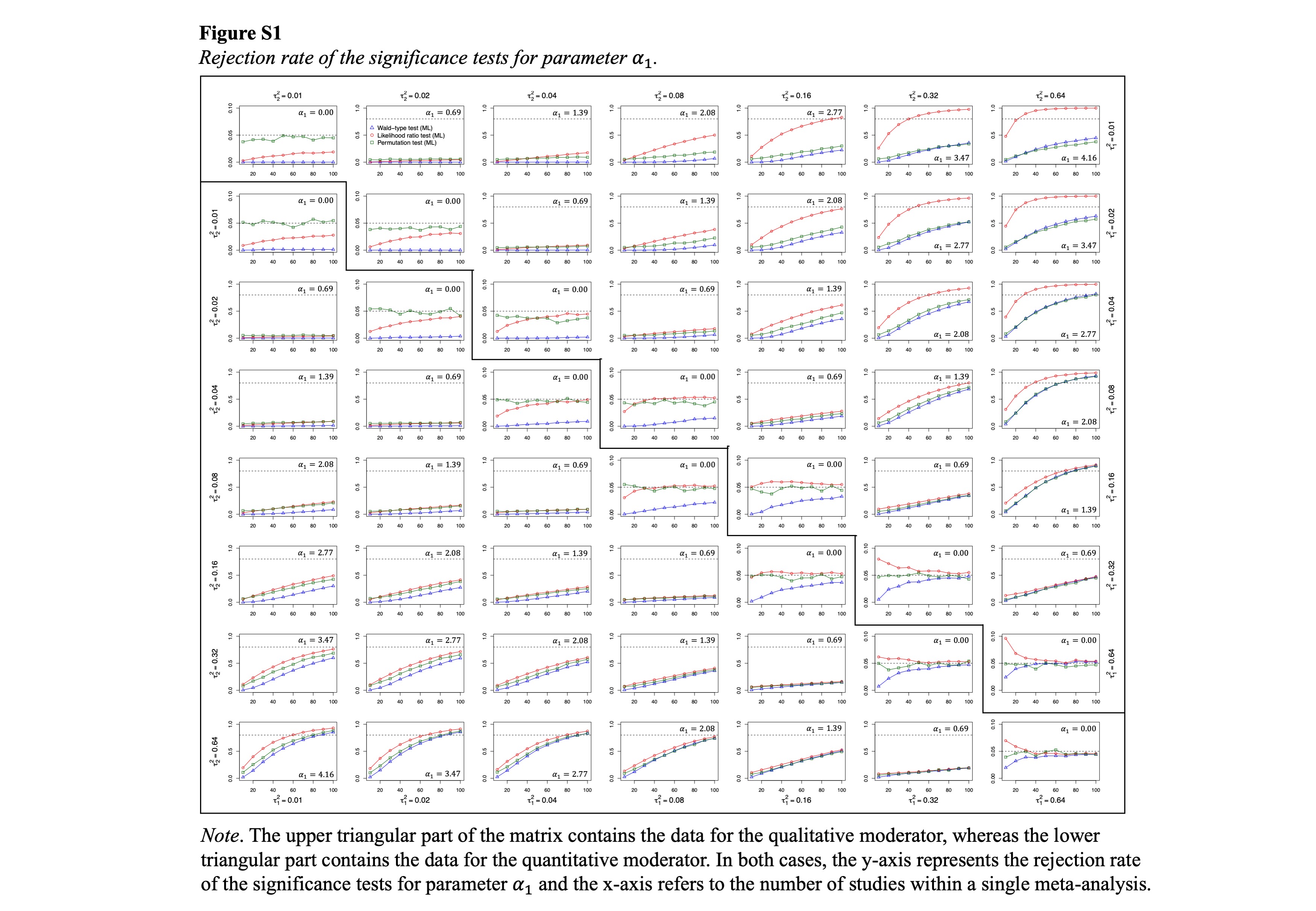

Supplement: Supplementary file 1 — Supplementary file1 (JPG 1094 KB) [file 13428_2025_2622_MOESM1_ESM.jpg]

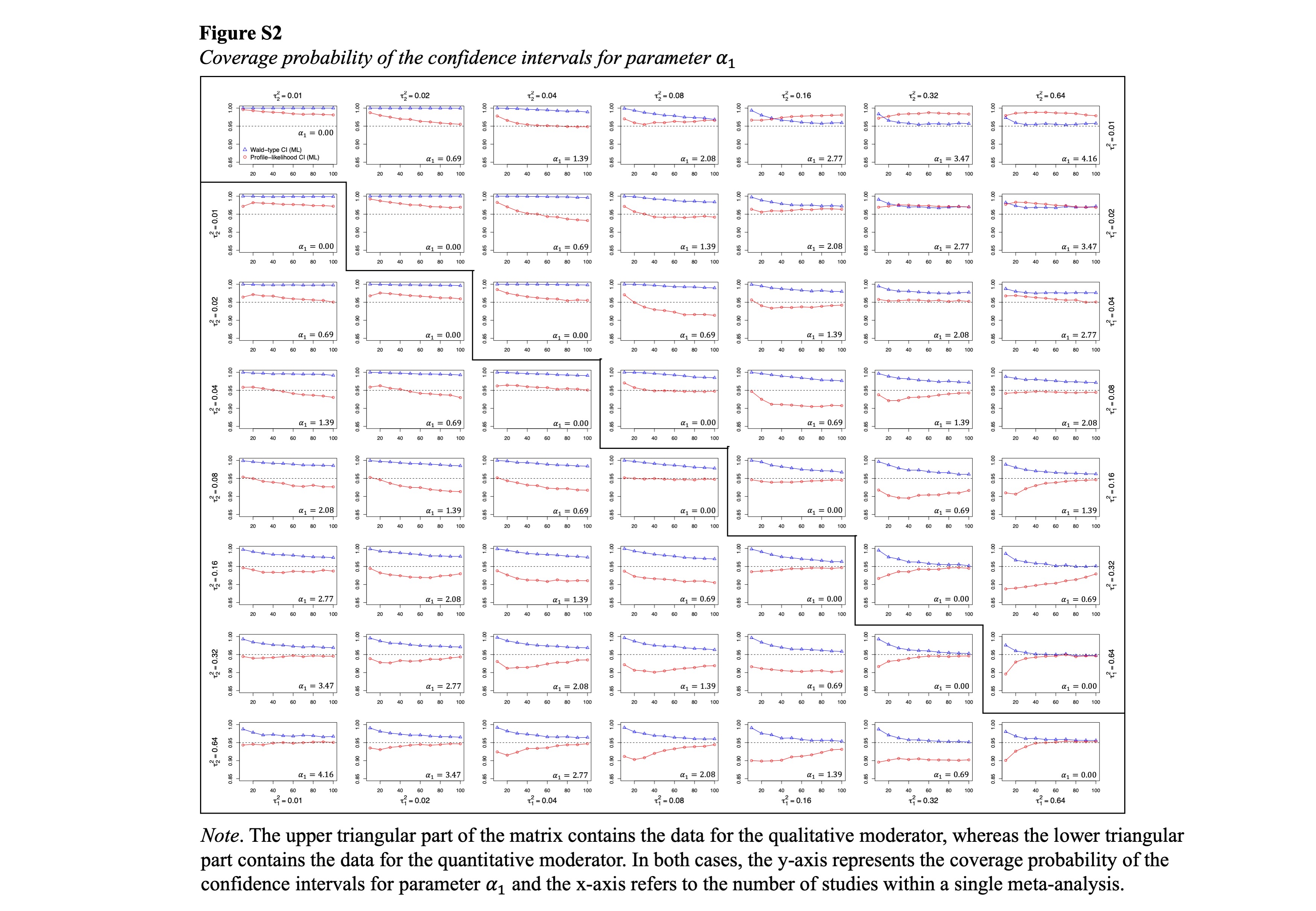

Supplement: Supplementary file 2 — Supplementary file2 (JPG 1079 KB) [file 13428_2025_2622_MOESM2_ESM.jpg]

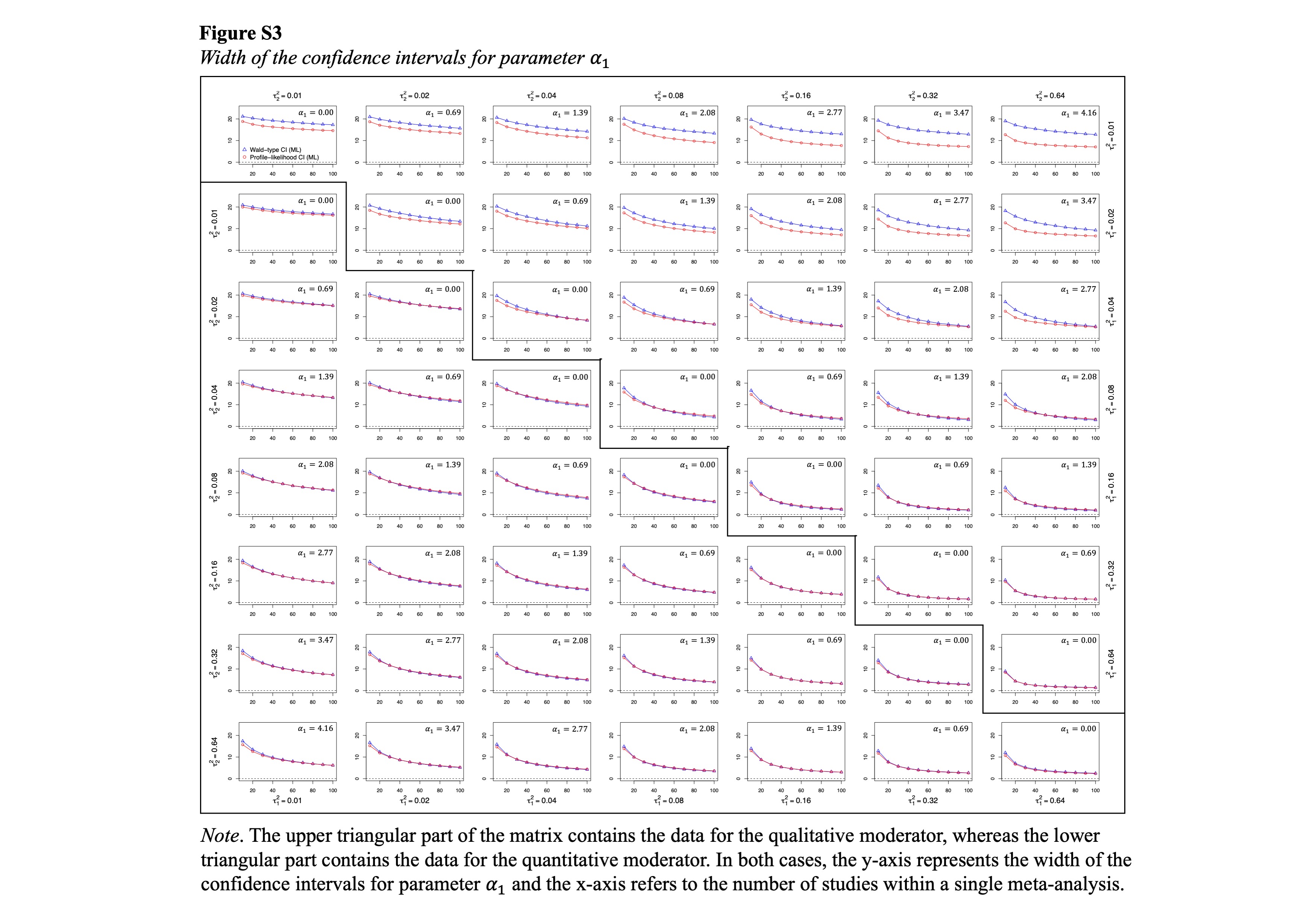

Supplement: Supplementary file 3 — Supplementary file3 (JPG 966 KB) [file 13428_2025_2622_MOESM3_ESM.jpg]

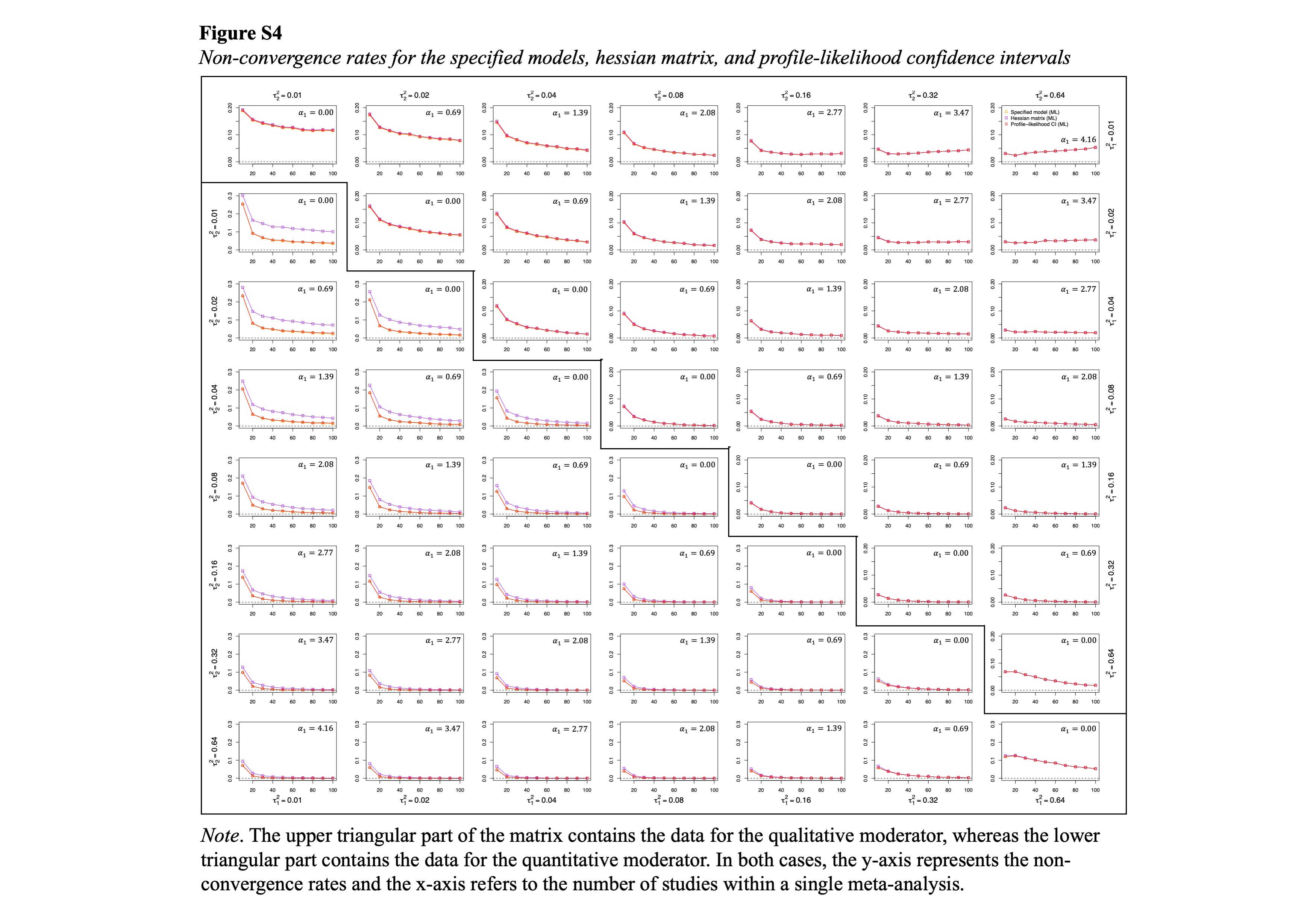

Supplement: Supplementary file 4 — Supplementary file4 (JPG 960 KB) [file 13428_2025_2622_MOESM4_ESM.jpg]
